# Biofortification of *Triticum* species: a stepping stone to combat malnutrition

**DOI:** 10.1186/s12870-024-05161-x

**Published:** 2024-07-15

**Authors:** Jitendra Kumar, Dinesh Kumar Saini, Ashish Kumar, Supriya Kumari, Vijay Gahlaut, Mohammed Saba Rahim, Ajay Kumar Pandey, Monika Garg, Joy Roy

**Affiliations:** 1 National Agri-Food Biotechnology Institute (NABI), Mohali-140306, Mohali, Punjab India; 2 Department of Plant Breeding and Genetics, Punjab Agricultural University, Ludhiana, 141004 India; 3 University School of Biotechnology, Guru Gobind Singh Indraprastha University, New Delhi, 110078 India; 4 Department of Biotechnology, University Center for Research and Development Chandigarh University, Gharuan, Mohali, Punjab 140413 India; 5 CSIR - Institute of Himalayan Bioresource Technology, Palampur, Himachal Pradesh 176061 India

**Keywords:** Biofortification, Wheat, Micronutrients, Malnutrition, Transporter proteins

## Abstract

**Background:**

Biofortification represents a promising and sustainable strategy for mitigating global nutrient deficiencies. However, its successful implementation poses significant challenges. Among staple crops, wheat emerges as a prime candidate to address these nutritional gaps. Wheat biofortification offers a robust approach to enhance wheat cultivars by elevating the micronutrient levels in grains, addressing one of the most crucial global concerns in the present era.

**Main text:**

Biofortification is a promising, but complex avenue, with numerous limitations and challenges to face. Notably, micronutrients such as iron (Fe), zinc (Zn), selenium (Se), and copper (Cu) can significantly impact human health. Improving Fe, Zn, Se, and Cu contents in wheat could be therefore relevant to combat malnutrition. In this review, particular emphasis has been placed on understanding the extent of genetic variability of micronutrients in diverse *Triticum* species, along with their associated mechanisms of uptake, translocation, accumulation and different classical to advanced approaches for wheat biofortification.

**Conclusions:**

By delving into micronutrient variability in *Triticum* species and their associated mechanisms, this review underscores the potential for targeted wheat biofortification. By integrating various approaches, from conventional breeding to modern biotechnological interventions, the path is paved towards enhancing the nutritional value of this vital crop, promising a brighter and healthier future for global food security and human well-being.

**Supplementary Information:**

The online version contains supplementary material available at 10.1186/s12870-024-05161-x.

## Background

Wheat (*Triticum spp*.) serves as a primary calorie source for a substantial part of the global population and ranks as the second most vital cereal crop worldwide [1–4]. Hexaploid bread wheat accounts for about 95% of global wheat cultivation, with the remaining 5% comprising tetraploid durum wheat [5]. In terms of cultivated area and food provision, wheat holds paramount importance as a major staple crop, contributing to 28% of the world’s edible dry matter and up to 60% of daily calorie intake in various developing nations [5]. Wheat products contribute approximately 20% of the protein and energy content in the average individual’s diet [4] Wheat grains comprise three main components: bran (also known as seed coat and is rich in fiber, B vitamins, and minerals), endosperm (contains carbohydrates, 15% lipids and 13% proteins like globulins, albumins, glutenins, and gliadins), and germ (nutrient-dense with vitamins, minerals, and healthy fats). The bran comprises 13–17%, endosperm 80–85% and germ 2–3% of wheat kernel. Nutritionally, wheat is a key source of carbohydrates and proteins, vitamins and minerals like magnesium; molybdenum; potassium; zinc; aluminium; copper; phosphorus; sulfur; Iron; and selenium, and several amino acids e.g., valine, asparagine, aspartic acid, alanine, glutamine, proline, methionine, glycine, phenylalanine, threonine, leucine, arginine, tryptophan, isoleucine, tyrosine, serine, histidine, and lysine [6, 7]. Hence, the composition and nutritional profile of wheat grains exert a significant influence on human health and well-being, particularly in developing regions. While the Green Revolution has undoubtedly enhanced agricultural productivity, the continuous decline in nutritional quality, particularly among high-yielding cereal crops including wheat, has been attributed to the intensified agricultural practices in the post-green revolution era [8]. However, the combined effects of domestication and associated evolutionary phenomena, such as the founder effect, resulting from modern breeding practices, have led to a reduction in the genetic diversity of crop species [3]. Domesticated wheat exhibits significantly lower levels of micronutrients and has limited genetic variation compared to its wild counterparts [9]. By employing hybridization techniques between cultivated and wild relatives, it becomes possible to reintroduce alleles that were lost during the domestication process, thereby enriching the cultivated gene pool [10] and enhancing the grain’s nutritional content [6, 10]. Notably, wild emmer wheat harbors a diverse array of alleles, offering substantial potential for improving the concentration of mineral nutrients including Fe, Zn, Se and Cu in cultivated wheat grains [10–12].

Micronutrients play a fundamental role in biochemical and physiological functions of biological systems. The world’s growing population exerts significant pressure on the global food system, with projections estimating an increase in human population from 7.6 billion in 2017 to 9.8 billion by 2050 [13]. In recent years, there has been a significant increase in malnutrition, which plays a substantial role in adding to the global disease burden. Specifically, pregnant women and children under 5 years of age face the most severe consequences of micronutrient deficiencies [2]. Recent data indicates that approximately 45% of deaths in children under the age of 5 are attributable to undernutrition on a global scale [13]. Nutrient deficiencies are often difficult to detect, hence the term ‘hidden hunger’ [2]. The World Health Organization (WHO) estimates that over two billion individuals across all age groups suffer from hidden hunger worldwide with a higher vulnerability of women compared to men [14, 15]. Despite the substantial efforts invested in this field, more needs to be done to adequately address the issue of micronutrient deficiency.

As of 2021, India ranked 101st out of 116 countries on the Global Hunger Index, indicating a significant challenge in addressing hunger and malnutrition (https://www.globalhungerindex.org/ranking.html accessed on 04 September 2022). To combat this issue, the India Biofortification Program was initiated in 2007 through collaborative efforts between HarvestPlus and the Department of Biotechnology (DBT), Government of India (https://www.ipcinfo.org/fileadmin/user_upload/sciencecouncil/Medium_Term_Plans/HARVESTPLUS_2009-2011_MTP.pdf; accessed on September 05, 2022). Biofortification is an enduring approach designed to combat micronutrient deficiencies by augmenting the concentrations of vital vitamins and minerals in staple crops that are often consumed by individuals in afflicted communities.

The program aimed to develop and distribute germplasm of various crops, including wheat, rice, and maize, biofortified with essential micronutrients such as iron (Fe), zinc (Zn), and pro-vitamin A. This program focused on utilizing micronutrient-enriched genotypes for crossbreeding purposes. Over the years, biofortified varieties of several crops have been released in approximately 30 countries, including pro-vitamin A-rich orange sweet potato, yellow cassava, orange maize, Fe-rich beans and pearl millet, and Zn-rich wheat and rice (http://www.harvestplus.org). Wild relatives and domesticated wheat species exhibit significant genetic diversity for these micronutrients, which have been effectively utilized to develop biofortified wheat varieties. As a result, 40 biofortified wheat cultivars have been released in different countries, including Bolivia, Bangladesh, Pakistan, India, Mexico, and Nepal [16]. Recently, in celebration of World Food Day, the Hon’ble Prime Minister of India, Mr. Narendra Modi, dedicated 17 biofortified crop varieties to the nation (https://icar.org.in/content/pm-dedicates-17-biofortified-varieties-8-crops-nation-0; accessed on October 17, 2020). The wheat varieties biofortified were Wheat ‘HI 1633’, MACS 4058‘, ‘DBW 303’’ for High Fe, Zn, and protein; ‘HD 3298’ for high iron only and ‘DDW 48’ for high protein.

Several recent reviews on wheat biofortification, including those [16–20], have contributed valuable insights into this field. However, there is an opportunity to enhance the existing literature by consolidating diverse information into a single, comprehensive resource. For instance, Kamble et al. [18] focused on wheat biofortification components such as Fe and Zn dynamics, genetics, and seed multiplication within the context of India. Gupta et al. [16] systematically examined genetic variations, QTLs, micronutrient accumulation, and emerging technologies, while Gupta et al. [17] provided an exhaustive review covering genetic variation, QTL identification, micronutrient physiology (refers to the study of how essential micronutrients are absorbed, transported, utilized, and regulated within the plant), and breeding approaches. Wani et al. [19] contributed genomic information related to Zn and Fe concentrations. However, even an edited volume, as noted by Ibba et al. [21], fell short of encapsulating all facets in a single chapter.

In contrast, the present review addresses this gap by offering a consolidated framework that encompasses diverse areas critical to understanding wheat biofortification. It provides a timely and necessary resource by covering aspects such as daily intake enhancement of essential minerals (Fe, Zn, Se, and Cu), exploration of genetic variability, identification of QTLs and MTAs associated with targeted micronutrients, insights into uptake and accumulation mechanisms, and a comprehensive discussion on various biofortification techniques. These techniques primarily include agronomic methods and genetic biofortification methods. Genetic biofortification encompasses a diverse range of approaches, including conventional breeding, transgenesis, molecular breeding, mutagenesis, metabolic engineering, and genome editing. Furthermore, the review explores the potential of expedited biofortification through speed breeding while thoughtfully addressing the challenges, limitations, and opportunities intrinsic to wheat biofortification. Through this comprehensive approach, the present review aims to serve as a singular and valuable resource for future researchers seeking a holistic understanding of wheat biofortification.

## Main text

### Improvement in daily intake of Fe, Zn, Se, and Cu by biofortified wheat

Excessive consumption of conventional wheat products exacerbates human micronutrient malnutrition due to their low essential micronutrient levels (viz., Fe, Zn, Se, Cu) and elevated anti-nutritional factors, limiting bioavailability [21–24]. However, the aleurone layer of wheat grains contains higher levels of micronutrients compared to the endosperm (starch). It is worth noting that the aleurone layer is often removed during processing. Thus, if conventional wheat products include the aleurone layer, there is a reduced likelihood of exacerbating malnutrition. Consequently, there exists a significant challenge and urgent need to enhance grain micronutrient concentrations and their bioavailability. According to recommended dietary allowances (RDA), the adult men and women group require approximately 10–15 mg/day of Fe, 9–15 mg/day of Zn, 0.055 mg/day of Se, and 1.0-1.6 mg/day of Cu [25]. Thus, adequate enhancement of these nutrients in wheat grains is essential for improving human health. Considering the above, biofortification emerges as a promising solution to increase micronutrient levels in staple crops, including wheat. Wheat is one of the foremost staple crops and contributes to the global food supply as it is the main ingredient in a number of food products e.g., bread, chapatti, cookies, porridge, breakfast cereals, pancakes, crackers, pastries, pasta, pizza, muffins and many more. Once developed, biofortified wheat can be one of the healthy future foods to combat malnutrition.

### Exploring genetic variability for Fe, Zn, Se and Cu

The concentrations of micronutrients such as Zn, Fe, Cu, and Se remained stable between 1845 and the mid-1960s. However, they have significantly decreased since then, coinciding with the introduction of semi-dwarf, high-yielding wheat cultivars [26–28]. Wheat landraces and wild relatives are the potential genetic resources that serve as a reservoir of natural variation for improving micronutrient concentration. Several studies have been undertaken over the last two decades to investigate the diversity of grain Zn, Fe, Se and Cu concentrations in wheat varieties and landraces [2–4,10, 29–95] . It was also demonstrated that during breeding for high-yielding semi-dwarf wheat cultivars following the green revolutions of the 1960s and 1980s, grain micronutrient levels decreased at the expense of higher grain yield [17, 28]. According to a study that examined genetic variability in 80 wheat genotypes, Zn and Fe levels have decreased by 0.13 mg/kg/year in absolute terms and 0.3% reduction/year in relative ones over the last 70 years [17, 28].

The accumulation of disproportionately more starch in the endosperm as a result of breeding for higher yield has been attributed to this reduction, colloquially known as the ‘dilution effect’. As a result, high-yielding genotypes have low amounts of Zn and Fe, while low-yielding genotypes have large levels of these micronutrients [28]. Zn and Fe variation has also been observed in diploid (2x) progenitors of tetraploid (4x) and hexaploid wheat (6x) [17]. The wild parents of synthetic hexaploid wheat (SHWs) had Zn and Fe levels 2–3 times higher than modern wheat and SHWs [17].

Some important studies [2–4,10, 29–94] reporting genetic variations for Fe, Zn, Se and Cu in wheat are presented in Additional file1; Table S1. Significant variations were found for grain Fe concentration (GFeC) (up to 88 mg kg^–1^) and GZnC (14 to 190 mg/kg) among wild wheat, especially wild emmer wheat (*Triticum turgidum* ssp. *Dicoccoides*) [16].

There are contradictory reports on the amount of genetic variability among the wheat genotypes and/or cultivars for the Se content/density in grains. Some of the studies have found no indication of genetic variability [95], while some studies have reported a higher Se density in wheat grains [17]. Recently, average content of grain Se content (GSeC) and GZnC reported in the 95 RILs of *Aegilops tauschii* was 10.89 mg/kg and 39.03 mg/kg, respectively [96]. In the absence of genetic variability for a trait in the available germplasm, mutagenesis is always a powerful strategy to create and broaden the genetic variation [17]. Kumar et al. [3] reported genetic variations in Fe and Zn contents among EMS mutant wheat lines, ranging from 26.20 to 49.90 and 23.08 to 46.5 ppm, respectively. The primary and secondary gene pool of wheat like *Aegilops tauschii*, *T. monococcum*, *T. turgidum dicoccoides*, *T. boeoticum*, *T. spelta* and *T. polonicum* have been reported to contain high variations for GFeC and GZnC contents [2–4,10, 29–94]. Therefore, these species stand as prominent sources for gene transfer to cultivated wheat varieties, facilitating the enhancement of micronutrients content. *T. turgidum* ssp. *dicoccoides* notably stands as a promising donor for GFeC and GZnC content, with GFeC ranging from 15 to 109 mg/kg and GZnC ranging from 14 to 190 mg/kg in different accessions [16]. Further, extensive backcrossing is generally required for direct transfer of genes from wild species to wheat. This can be avoided by using synthetic amphiploids and alien addition/substitution lines Gupta et al. [17].

### Mapping QTLs, meta-QTL analysis and genes regulating Fe, Zn, Se and Cu content or concentration

Numerous QTL mapping studies have been conducted to identify loci associated with high grain concentrations of Fe and Zn across all 21 chromosomes in various types of wheat, including diploid, tetraploid, and hexaploid species [97]. Recently, a QTL mapping study by Liu et al. [55] utilized a RIL population of 200 lines derived from a cross between a Chinese parental line and a Mexican commercial bread wheat cultivar and identified a total of 60 QTLs associated with agronomic-related traits, grain protein content (GPC), and micronutrients. Among these, ten QTLs were associated with grain GZnC and nine QTLs with GFeC. Leveraging the availability of the annotated wheat genome sequence, the authors further identified 55 promising candidate genes associated with GFeC and GZnC. In some of the QTL mapping studies, a significant positive correlation between GZnC and GFeC has been observed, suggesting the possibility of co-localization of QTLs or a multi-trait effect influencing the concentrations of both Zn and Fe in wheat grains [49]. The presence of these co-localized QTLs is pivotal for concurrently enhancing Fe and Zn levels, making them crucial for effective breeding initiatives.

In a recent study, Rathan et al. [65] conducted QTL mapping, pinpointing genomic regions linked to GFeC and GZnC in a wheat bi-parental mapping population across three environments. The study unveiled four GFeC and nine GZnC QTLs consistently observed in at least two environments, spread across chromosomes 1D, 1B, 2 A, 2B, 5 A, 6B, and 7D. Furthermore, another study reported a total of 23 QTLs for GFeC and 27 QTLs for GZnC across multiple environments. In a similar way, Wang et al. [66] reported seven QTLs for GZnC and four QTLs for GFeC, distributed on chromosomes 1D, 2 A, 3B, 3B, 4D, 6 A, 6D, and 7B, respectively. Notably, the QTLs located on chromosomes 4D, 6 A, and 7B exhibited pleiotropic effects on both grain Zn and Fe concentrations. Additionally, QTL analysis conducted on a RIL population (HD3086 x HI1500) identified nine QTLs for GFeC and 11 QTLs for GZnC [98].

Very few QTLs have been reported for GSeC in wheat as compared to GZnC and GFeC. These QTLs have been mapped on different chromosomes such as 1 A, 1B, 2B, 3 A, 3D, 5 A, and 5B, as well as homoeologous groups of 4 and 6 chromosomes [98]. For instance, Yan et al. [12] reported six QTLs for GSeC, these QTLs were mapped on six different chromosomes such as 1 A, 1B, 3 A, 5 A, 7 A, and 7B with PVE up to 18.6%. Pu et al. [40] reported QTLs for GSeC on chromosome 3D and 5 A with contributions to phenotypic variation up to 28.38%. Similarly, Wang et al. [91] identified two major QTLs for GSeC on chromosome 3D and 5 A. Further, two QTLs controlling GSeC and GZnC content were identified on 1D and 2D chromosomes, respectively, that explained 11.94% and 13.49% of the phenotypic variations, respectively [96].

To best of our knowledge, only three mapping studies have reported QTLs for GCuC in wheat so far. Peleg et al. [10] used a population of 152 F_6_ RILs, developed by single-seed descent from a cross between durum wheat and wild emmer wheat and identified a total of 10 QTLs having PVE values ranging from 0.7 to 13.1% and located on different chromosomes viz. 1 A, 2 A, 3B, 4 A, 4B, 5 A, 6 A, 6B, 7 A, 7B. Using a DH population derived from winter wheat Hanxuan 10 and Lumai, a total of 6 QTLs having PVE values ranging from 5 to 15.7% were mapped on 2 A, 4 A, 4D, 5 A, 6 A, and 7B [38]. In another study conducted by Pu et al. [40], using a population of 171 RILs derived from SHW-L1 x Chuanmai 32, a total of 8 QTLs (PVE ranging from 4.8 to 12.7%) were identified and mapped on 2 A, 3D, 4 A, 4D, 5 A, 6D and 7B. Using a RIL derived from ‘CO960293-2’/‘TAM 111, Yu et al. [99] identified five QTLs for three grain micronutrients including GCuC and mapped them on 3B, 5A, and 7B. Detailed information on QTLs available for Fe, Zn and Se are provided in Additional_file_2; Table S2.

Further, the associations or co-localizations between QTLs, governing various micronutrients, may be linked to the physiological coupling of processes that regulate mineral accumulation in grains. It is crucial not to overlook genomic regions controlling individual minerals, as they likely play roles in other mineral-specific mechanisms. Thus, the simultaneous identification of QTLs for multiple minerals is advisable. This approach facilitates the dissection of intra and interrelationships, offering insights into the functional basis of genomic architecture, physiology, and evolution in the system of mineral accumulation in wheat grains or other parts. However, previously known QTLs associated with various traits, including micronutrients in wheat, often lack the robustness needed for significant use in MAS within wheat breeding. Hence, there has been a recent shift towards meta-QTL (MQTL) analysis for nearly all crucial traits in wheat, including quality traits related to micronutrients [100, 101]. Concurrently exploring multiple QTLs associated with a single trait or a related trait across diverse populations and environmental conditions aims to identify hot spot regions and stable MQTLs in the plant genome. In a 2022 study, Gudi et al. [100] identified 11 most stable and reliable MQTLs (each having large PVE values, reduced CIs, and involved a large number of QTLs) associated with various quality traits, including different micronutrients. Similarly, in another study, Singh et al. [101] identified three potential MQTLs associated with both Fe and Zn contents, exhibiting high PVE values and reduced CIs. These MQTLs have been recommended for use in breeding programs aimed at biofortifying wheat.

Additionally, through expression analysis, a few genes encoding (a) heat shock protein, (b) carotenoid oxygenase, (c) FAD-linked oxidase, and (d) HRZ containing zinc finger, RING/FYVE/PHD-type domains have been validated for their association with Fe and Zn homeostasis in wheat. These genes can be employed to understand the molecular basis of genetic Fe and Zn homeostasis and utilized for marker development and fine mapping, serving as valuable tools for MAS [101]. Homeostasis refers to the process of maintaining an optimal level of redox-active metals in plants. This is achieved through the balanced activities of transporters that facilitate the import of metals into the cell, their proper distribution to where they are needed, storage within the cell, and utilisation of metalloproteins and metalloenzymes [102]. Se and Cu homeostasis in wheat also involves a complex network of genes that regulate uptake, assimilation, and distribution within the plant which can be understood by the mechanism of mineral uptake and translocation (please refer to mechanisms of mineral uptake and translocation portion of this review).

### Genome-wide association studies: revealing genomic regions associated with grain Fe, Zn, Se, and Cu contents

Identifying significant markers or marker-trait associations (MTAs) and potential candidate genes via GWAS may assist breeding for increasing micronutrient concentrations in wheat varieties. Therefore, several GWAS studies have been conducted in wheat for the identification of MTAs associated with micronutrient concentrations. For instance, Manickavelu et al. [45] used 269 Kihara Afghan wheat landraces for GWAS analysis and detected one marker (1,208,679|F|0Ð64:T > C) on 6D significantly associated with Zn in wheat. Later, Gorafi et al. [51] identified three marker loci associated with Zn content on 1D, 2D, and 5D and three marker loci associated with Fe content on 2D, 4D, and 5D. In the same year, three other studies were also published. The first study identified a total of 92 MTAs associated with ten minerals (viz., Fe, Zn, Ca, Cd, Cu, Co, Li, Mg, Mn, and Ni) in the grains of 123 synthetic hexaploid wheat lines. Of these 92 MTAs, 60 were novel, 40 were within genes, and the genes underlying 20 MTAs had annotations signifying a potential role in mineral concentration in grains. Furthermore, they identified multi-traits stable MTAs and recommended top 13 synthetic hexaploid wheat lines having higher concentrations of important grain micronutrients, including Zn, Fe and Cu, for utilization in the breeding program for genetic biofortification of wheat [52]. Using single-locus single-trait GWAS approach, 584 significant MTAs were detected for four nutritional traits (viz., Zn, Fe and beta-carotene contents, GPC) at two locations; of these 584 MTAs, only ten MTAs could pass Bonferroni correction. Using the multi-locus mixed model of GWAS, they identified 271 MTAs (after Bonferroni correction) for the same four nutritional traits. Furthermore, they also successfully mapped a total of 73 epistatic interactions involving 146 markers {spread over 18 of the 21 chromosomes (except 4D, 5D and 6D). Epistasis is generally overlooked in GWAS, yet understanding epistasis is crucial for comprehending the complex genetic architecture of a trait [2].

The HarvestPlus Association Mapping (HPAM) panel, consisting of 330 bread wheat lines and genotyped with the Illumina iSelect 90 K Infinitum SNP array, was utilized to identify 39 MTAs for grain Zn. Two significant effect QTNs were detected on chromosomes 2 and 7, and candidate genes, mainly for metal ion binding and Zn finger motif of transcription factors, were also characterised in these QTN regions [79]. Recently, Arora et al. [53] detected 19 MTAs associated with different micronutrients in grains of *Ae. tauschii* accessions. Out of these 19 MTAs, five, four and three significant associations (after Bonferroni correction) were detected for Fe, Zn, and Cu, respectively. These associations were linked with genes encoding transcription factor regulators, transporters, and phytosiderophore synthesis. Recently in 2020, using the HPAM panel consisting of 330 wheat lines, Cu et al. [59], detected 279, 379 and 481 significant MTAs for five nutrient concentrations (viz., Fe, Zn, Cu, Mn, and P) in mature grain, immature grain, and immature rachis, respectively. In addition, 116 potential candidate genes linked to key MTAs were identified on 1 A, 2 A, 3B and 5B using the annotated wheat reference sequence (RefSeq V1.0). Most of these identified genes were grouped into five different groups based on their putative functionality. These groups were: (i) transporter families including Zn transporter such as yellow stripe-like (*YSL*) 1, 9 and 12 transporters, vacuolar iron transporter (*VIT*), Cu, potassium, sulfate, phosphate and sugar transporters, (ii) protein processing enzymes, (iii) storage proteins (iv) metallo-protein and (v) antioxidant enzymes [59]. Further, twenty-three QTNs were identified for GCuC, and these were mapped on different chromosomes such as 1 A, 1D, 2 A, 2B, 2D, 3 A, 3B, 3D, 4 A, 4B, 4D, 5 A, 6D, 7 A, and 7B, with PVE 2.6–5.8%. Sixteen QTNs were detected for GZnC, and these were mapped on different chromosomes like 1B, 2B, 2D, 3 A, 3D, 4 A, 4B, 5 A, 5D, 6B, and 7D with PVE 2.7 ~ 6.6% by GWAS using two hundred and forty-six wheat varieties. Furthermore, five pleiotropic QTNs were also found; these QTNs were mapped on 2B, 2D, 3 A, 4B, and 5 A [63]. Six high-confidence MTAs for GFeC and three for GZnC were identified. These MTAs were successfully mapped onto chromosomes 1 A, 2 A, 3B, 4 A, 4B, 5 A, and 7B, utilizing a dataset of 161 advanced lines derived from wild emmer. Additionally, 34 CGs were also identified. These CGs encode significant proteins/products that play crucial roles in various biological processes, including catalysis, transporter proteins, transcription factors, and proteins involved in defense mechanisms [63]. More recently, 4 MTAs for Fe and 2 for Zn content were reported using an association panel involving both old and new Indian elite varieties [65]. In addition, five MTAs for each Fe and Zn were reported in advanced breeding lines and commercial cultivars of wheat [59]. Similarly, 254 genomic variants were identified for Fe and Zn in the CGs through Comparative genome analysis [68]. Further, nine stable QTLs for GZnC were identified on chromosomes 3 A, 4 A, 5B, 6D, and 7 A using 382 wheat accessions in three different environments [4]. The transfer of these MTAs/QTLs/QTNs through marker-assisted breeding will undoubtedly enhance mineral concentrations in wheat grains. However, detecting QTLs with major effects and stable expression across environments has been a constraint for marker-assisted breeding. Moreover, discrepancies commonly arise when comparing GWAS studies for the same trait, potentially stemming from variations in allele frequency between populations, insufficient control of population structure, or environmental factors. Data from the same environment(s) share a common environmental component, but merging multiple panels grown under different conditions can lead to inaccuracies in assigning environmental effects to genetic variations across the panels [103]. Meta-GWAS may offer a more effective solution to address these challenges, allowing for the use of data from independent studies and employing available statistical methodologies [104]. MTAs identified through meta-GWAS hold substantial promise for breeding programs.

### Genomic predictions for Fe, Zn, Se, and Cu concentrations

An advanced form of MAS, i.e., genomic prediction (GP) or genomic selection (GS) or genome-wide selection (GWS), has been proposed, which relies on genome-wide markers information to predict the breeding values of complex traits in the populations (known as breeding or test populations) for which only genotypic data are provided. These predicted breeding values are called genomic estimated breeding values (GEBVs) and are based on both phenotypic and genotypic data from the individuals in a training population. Numerous methods have been adopted for GP or GS calculation such as Bayesian methods, rrBLUP and genomic best linear unbiased prediction (GBLUP) [105]. Applying GS approach can speed up genetic gains in biofortified wheat development. Costly and tedious mineral concentration phenotyping can be bypassed by using GS, boosting genetic gain per generation via early selection. Velu et al. [106] published the first study where they assessed the accuracy of GPs of elemental contents in wheat. Prediction abilities estimated using GP models under different environments ranged from 0.331 to 0.694 for Zn and from 0.324 to 0.734 for Fe concentration. In another recent study, GP was evaluated for Fe concentration in grains with three statistical models, including GBLUP, ridge regression best linear unbiased prediction (RR-BLUP), and Bayes-Cπ. Prediction ability values were 0.29 to 0.38, 0.27 to 0.35, and 0.20 to 0.35 based on three models used, respectively [80]. In a more recent study conducted by Meher et al. [107], the effectiveness of eight Bayesian genomic prediction models was evaluated for predicting three micronutrient contents, which included Fe and Zn contents. The study analyzed training sets with both 90% and 50% genotypes. The results indicated that the genomic prediction accuracy for GFeC increased from 0.088 to 0.407, and for GZnC, it increased from 0.47 to 0.67.

### Mechanisms of mineral uptake and translocation

**Fig. 1:**
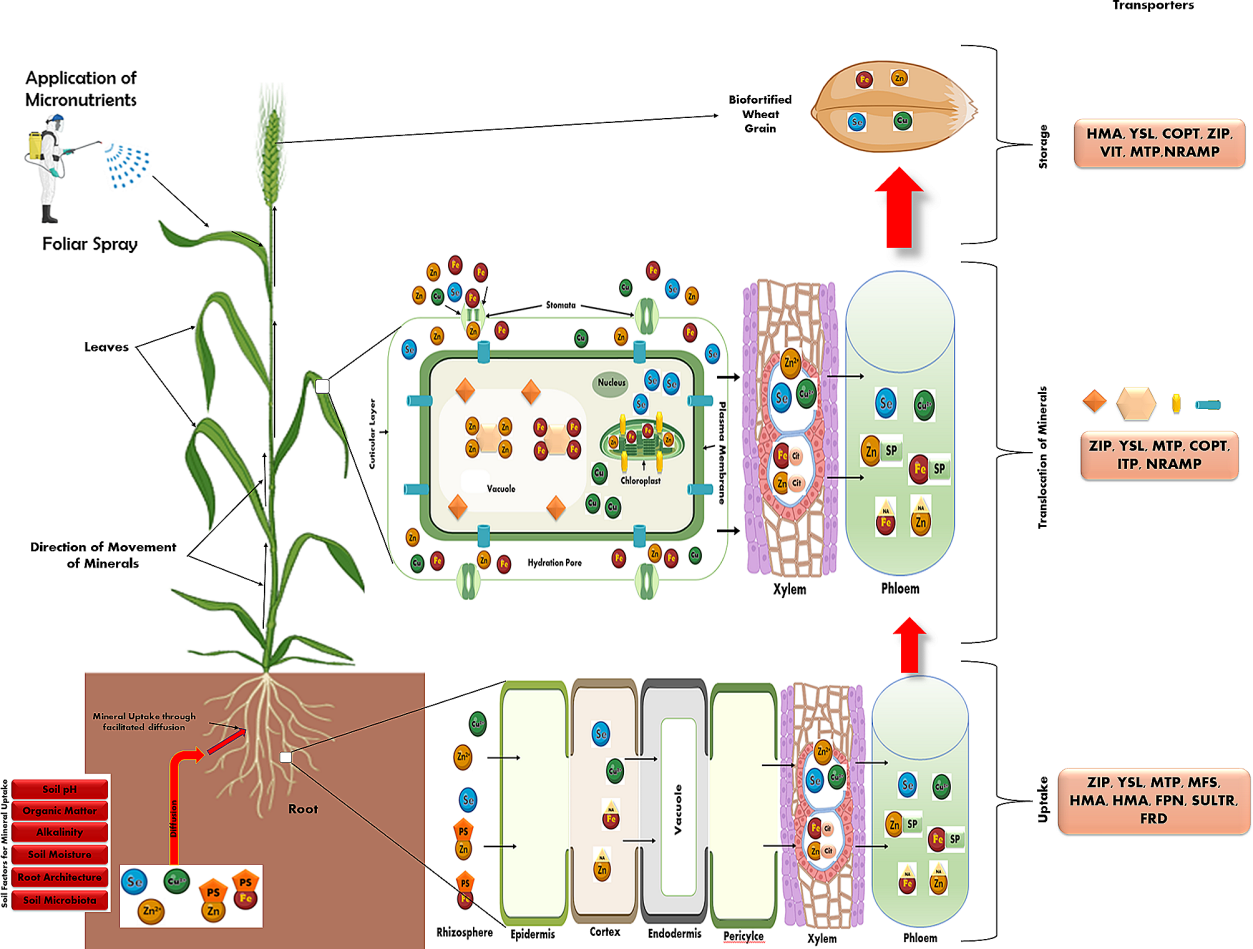
A Simplified proposed pathway for Fe, Zn, Cu and Se uptake and translocation to the grain in wheat: Putative classes of transporters depicted in the picture are based on evidence from other species. (ZIP = ZRT-IRT-like protein, YSL = yellow stripe-like transporter, MFS = major facilitator superfamily transporter, MTP = metal tolerance protein, HMA = heavy metal ATPase, FPN = ferroportin, NRAMP = natural resistance-associated macrophage protein, VIT = vacuolar iron transporter, ITP = Ion transport protein, OPT = oligopeptide transport family, SULTR = Sulfate transporter, COPT = copper transporter, PS = phytosiderophores, NA = nicotinamide, SP = small protein and Cit = Citrate

A complete understanding of the physiology and molecular basis of uptake, transport and storage of different micronutrients such as Zn, Fe, Se and Cu is a pre-requisite for biofortification in any crop including wheat. The uptake of micronutrients includes acquisition from the rhizosphere and their radial transfer to the xylem of the root. At the time of grain filling period, the micronutrients are transported to the grain via shoot [17, 108]. In this review, different aspects of physiology and molecular basis of biofortification, including uptake, translocation and storage of micronutrients, and their transporters are discussed (also depicted in Fig. 1).

### Zn uptake and translocation

Wheat employs two mechanisms to absorb Zn^2+^ ions. Initially, zinc ions (Zn^2+^) are absorbed across the root cell membrane through an active and selective transport mechanism that is driven by photosynthetic energy. Furthermore, in situations where there is a scarcity of zinc (Zn), the roots release complexing agents, such as MA, which bind to Zn, thereby enhancing its concentration and mobility within the soil solution. When the availability of Zn is low in soil, the role of soil moisture is very important [109]. The major determining factor of Zn availability for uptake is the pH of soil, which ultimately affects the solubility of Zn in soil solution. Lower soil pH enhances Zn adsorption to the cation exchange sites of soil constituents (like metal oxides) which ultimately reduces Zn availability in soil solution [109]. Mazhar et al. 2023 [110] studied Zn uptake and translocation in wheat and rice using ZnO nanoparticles in normal and salt stress conditions. Zn is taken up by plants mainly in the form of the bivalent cation Zn^2+^. Many families of proteins are vital to meet the requirements for homeostatic adjustment such as (i) the yellow stripe-like (YSL) family of proteins which are related to Zn transport to reproductive tissues and leaves [111]; transcriptome analysis has also been used to identify 67 YSL genes in wheat for Fe. YSL proteins facilitate long-distance transfer from leaves to grain through both xylem and phloem. They are also found in shoots, albeit to a lesser extent than in roots [112]. Observations of Fe uptake suggest that the YSL transporter family in wheat may also control the uptake of Zn from soil through the formation of a Zn-MA complex. Nevertheless, the specific members of the YSL transporter family that are accountable for the absorption of the Zn-MA complex have yet to be determined [113]. Plant shoot YSL transporters may also play a role in the Zn^3+^/Fe^3+^-PS complexes’ long-distance transit from leaves to grains via xylem and phloem [84], (ii) metal tolerance proteins (MTPs) that are involved in the storage of Zn in vacuoles. Twenty *TaMTPs* have been reported in wheat; these included *TaMTP1s* and *TaMTP8*s associated with Zn-CDFs, *TaMTP2s* associated with Fe/Zn-CDFs, and *TaMTP3-7s* associated with Mn-CDFs [114]. According to a different study, *TuMTP1* (*Triticum urartu* MTP1) may retain Zn^2+^ and Co^2+^ homeostasis by sequestering excess cytosolic Zn^2+^ and Co^2+^ into yeast vacuoles [114], (iii) Natural Resistance Associated Macrophage Protein 4 (*AtNRAMP4*), responsible for the root-shoot translocation of Zn, is among the NRAMP genes found in Arabidopsis. Other NRAMP genes, such as *AtNRAMPs*, *AtNRAMP1*, *AtNRAMP3*, etc., play roles in the transport of various metals [115], (iv) heavy-metal ATPases (HMAs), which are involved in plant tolerance to heavy metals and also in the transport of Zn and Cu to the vacuole [111]. After BLASTP searches a total of 27 HMAs were identified in wheat [116] and (v) Plant Cadmium Resistance2 (PCR2) which is related to the long-distance transport of Zn [111, 117, 118]. The entry of Zn into roots is mediated by nicotianamine, which forms complexes that are then carried to the vascular tissues. More information regarding Zn ligands and binding proteins, as well as particulars of transporter proteins including ZIF1 Like (ZIFL; Zinc-Induced Facilitator) and MTPs have been provided in some previous reviews [111, 117, 118]. Zinc (Zn) forms complexes with organic molecules (ligands) that share one or more electrons, especially those with N-, S-, and O-electron donors. Therefore, when aiming at increasing the concentration of Zn in wheat grain, it is important to take into account its chemical speciation. These compounds include non-proteinogenic amino acids such as: nicotianamine (NA) [2(S),3′(S),3″(S)-N- [N(3-amino-3-carboxypropyl)-3-amino-3-carboxypropyl] azetidine-2-carboxylic acid]; compounds derived from NA; the mugineic acid (MA) family phytosiderophores (PS) and 2’-deoxy mugineic (DMA) ; amino acids including histidine and cysteine; organic acid-carboxylates including malate and citrate; peptides; and small proteins (metallothioneins) [113].

Further, Durmaz et al. [119] published one study where they identified and cloned a full-length ZIP1 transporter (*TdZIP1*) in wild emmer wheat (*T. turgidum* ssp. *dicoccoides*) and also analyzed the corresponding protein sequence for structural attributes. Several ZIP genes including *OsZIP1*, *OsZIP3*, *OsZIP4*, *OsZIP5*, and *OsZIP8* were already characterized in rice for transport of Zn and out of these *OsZIP4*, *OsZIP5*, and *OsZIP8* were involved in response to Zn-deficiency in rice [120]. Furthermore, *OsZIP9* is localized to the plasma membrane and contributes to Zn uptake under Zn-limited conditions, particularly in soil [121]. The role of ZIP proteins in Zn assimilation and distribution in plants is summarised in a recent review by Lira-Morales et al. [122]. Recently, Deshpande et al. [123] observed a significant either positive or negative correlation between Zn/Fe-Regulated Transporter-Like Protein (ZIP) family (viz., *ZIP1*, *ZIP7*, *ZIP15*), *CA* (carbonic anhydrase), and *DMAS* (2’-deoxymugineic acid synthase) in flag leaves with Zn content in the grains depending on its developmental stages. They observed diverse gene expression among members of the ZIP family, with the specific cultivar type mostly associated with the Gpc-B1 gene [123]. In order to maintain metal homeostasis, a sophisticated control system permits the absorption, dispersion, and accumulation of these ions. In situations where metal ions are scarce, the uptake of these ions triggers the activation of many transport proteins, including ZIP family. These days, protists, animal fungus, plants, and bacteria have all been shown to contain members of the ZIP family. In hexaploid wheat, seven groups of F *TabZIP* genes and fourteen ZIPs with homeologs have been discovered. Furthermore, they provided a better understanding of Zn-homeostatic mechanisms in wheat, representing an expanded repertoire of group F bZIP TFs, adding to the complexity of Zn homeostasis. Similarly, in the roots of zinc-deficient wheat plants, the ZIP family transporters *TaZIP3* and *TaZIP7* are activated, enhancing both the absorption of zinc and the transport of zinc from the root to the shoot. The Zn loading from leaves into the phloem and Zn transport from the endosperm cavity into the modified aleurone layer, the aleurone layer, and finally the wheat endosperm is mostly regulated by ZIP family transporters [113, 124]. However, additional research is necessary to comprehensively understand the various members of the ZIP family that participate in zinc uptake in soil with low levels of zinc.

### Fe uptake and translocation

The availability of Fe depends upon pH of soil and its redox potential. Fe is readily oxidised at higher pH, forming insoluble ferric oxides. On the contrary, at low pH levels, the ferric (Fe^3+^) is freed from the oxide, so readily available for uptake by roots. Three strategies are there for Fe uptake, (i) strategy I, also known as reduction strategy; (ii) strategy II or chelation strategy; and (iii) strategy III a combination of both [125, 126]. Wheat predominantly uses the chelation strategy (strategy II) for Fe uptake from the soil, therefore from here onwards, we will only discuss strategy II.

The key step in strategy II is the production of Fe chelators, i.e., the mugineic acid (MA) family phytosiderophores (PSs). These PSs are produced from S-adenosyl-L-methionine via a series of conserved reactions catalyzed by Nicotianamine synthase (NAS), Nicotian Amine Amino Transferase (NAAT) and Deoxy Mugineic Acid Synthase (DMAS; Fig. 1) [127]. In wheat, a total of 21 NAS genes have been identified [128]. Moreover, DMA is produced by DMAS, and the TOM1 transporter subsequently releases DMA into the rhizosphere for Fe acquisition [126]. Besides, three *TaDMAS1* homeologs and six *TaNAAT* homeologs have also been identified in wheat that are most closely related to the barley *HvNAAT* and *HvDMAS1*. Fe uptake and translocation process in wheat is almost the same as in rice and has been previously reviewed [129].

A recent study by Sharma et al. [118] provided a comprehensive analysis of wheat ZIFL (Zinc-Induced Facilitator-Like) genes, identifying fifteen putative *TaZIFL*-like genes located on chromosomes 3, 4, and 5. This study also explored that the promoters of wheat ZIFL genes contained various metal binding sites, including those important in Fe and heavy metal homeostasis. Additionally, this study laid the groundwork for further investigation into the functional evaluation of candidate ZIFL genes as potential transporters of mugineic acid (TOM) proteins in wheat, as TOM is upregulated in wheat roots under Fe deficiency [130]. Moreover, transcriptome analysis identified eleven F-box and twelve NBS-LRR disease-resistance protein genes, which may also play a role in the regulation of Fe and Zn homeostasis in wheat [111].

### Se uptake and translocation

In cereals, selenomethionine (SeMet) is the predominant organic form of Se. Among cereals, wheat has demonstrated high efficiency in accumulating Se [131]. Currently, there is a lack of identified Se absorption mechanisms in plants due to the non-essential nature of Se as an element for plants [132]. Meanwhile, because Se and sulphur (S) have similar chemical characteristics, the absorption of Se(VI) follows the same route as sulphate. This is mostly facilitated by the SULTR1;1 and SULTR1;2 transporters through an active transport mechanism [133]. The phosphate transporter Pht1 family utilises aquaporins to uptake Se (IV) as HSeO_3_^−^ by roots [134, 135]. However, the uptake, and translocation of Se can vary among different plant types and cultivars [136]. Se is generally taken up by plant roots and moved via xylem to storage parts, leaves, and then via phloem to grains i.e. wheat [137–140]. Following foliar Se administration, selenium can also reach the leaves by passing through the cuticle or through the stomatal channel [141]. After that, it is moved to the edible portions of the plant, albeit this relocation is dependent on the plant’s phenological stage and nutritional state [141, 142]. Se can be applied in the form of selenite or selenate through foliar and soil ways to increase Se content in different parts of wheat [143]. Plant uptake of selenite, which is chemically similar to sulfate, is mediated by various sulfate transporters. The selectivity of these transporters for selenite and sulfate can differ based on nutritional status and plant species [136]. Moreover, within a single plant, different sulfate transporters may exhibit varying preferences for sulfate and selenate. A detailed understanding of the molecular-level selectivity of these sulfate transporters could contribute to the development of wheat genotypes biofortified with Se.

Initially, it was believed that selenite uptake by plant roots was not metabolically dependent [136]. Selenite is limited in its transfer to shoots and is rapidly absorbed into organic forms in roots. Selenate, on the other hand, is quite mobile in xylem transport but is not easily assimilated into organic forms. However, it was demonstrated by Li et al. [144] that selenite uptake in wheat could be suppressed by the metabolic inhibitor carbonyl cyanide m-chlorophenylhydrazone (CCCP). The CCCP is a protonophore and an uncoupler of oxidative phosphorylation, which causes a degeneracy of the proton motive force across the membranes, inhibited by phosphate in the nutrient solution, and enhanced by phosphorus deficiency. The excessive application of phosphate fertiliser has been observed to have a significant impact on the absorption of selenium (Se) by crops. This is primarily due to the similar chemical and physical properties of phosphate and selenite. Specifically, it has been found to inhibit the transport of Se from the root to the shoot [145–148]. However, the understanding of how phosphorus supply affects the subcellular distribution and chemical forms of Se is still limited [137–144]. A comprehensive review of Se uptake and translocation mechanisms in higher plants has been conducted by Zhu et al. and Trippe et al. [149, 150]. Recently, Wu et al. [151] conducted high-throughput transcriptomic sequencing in *Aegilops tauschii*, providing valuable insights into Se metabolism in this species for future research. Similar other recent studies can be conducted in wheat to gain a deeper understanding of the molecular and physiological mechanisms involved in Se metabolism [136, 151, 152].

### Cu uptake and translocation

Different oxidation states of copper can be found in soils; in acidic soils, copper is primarily found in the divalent form (Cu^2+^), but it can also be found in the monovalent form (Cu^+^) and even reduced form (CuO). Several transporter and transcription factors are involved in Cu homeostasis, including uptake, distribution, and accumulation [153]. The Cu ions are transported in the vasculature in the form of Cu^+^-metallothioneins or Cu^2+^-complexes. The homeostasis of Cu is vital for the normal growth and development of plants [153]. Cu transporters (COPT), known to transport the Cu into and within cells of plants, are mainly divided into two groups, Ctr, and P1B-type ATPase. These Cu transporters have specific structural characteristics, such as trans-membrane (TM) domains and conserved Cu transport-related motifs [153]. Ctr proteins have a central domain of three TM regions and are considered high-affinity Cu transporters. Most of these proteins contain specific motifs important for Ctr function, such as a Mets motif (methionines arranged in MXXM or MXM) at the N-terminus and a conserved MXXXM motif in TM2 [154]. Membrane-bound P1B-type heavy-metal ATPase (HMA) aids in the delivery of Cu into the target organelle or re-translocation to the apoplast once the Cu enters into the cytosol [155]. P1B-type Cu transporters possess six to eight TM domains and a large loop between TM6 and TM7 and have conserved metal-binding sites at the N-terminus [156]. Several HMAs were also identified in different plant species such as 31 in *Brassica napus*, 36 in *Solanum tuberosum*, 11 in *Zea mays* and 17 in *Populus trichocarpa*, 20 in *Glycine max*, 32 in *T. aestivum*, 14 in turnip landraces. Recently, Batool et al. [157] identified *TaHMA7, TaHMA8, and TaHMA9* genes for Cu homeostasis. In *O. sativa*, *OsHMA3* and *OsHMA4*, located at the tonoplast, regulate the accumulation of Cu and Cd in both seeds and roots. *OsHAM9*, a Cu efflux protein situated in the plasma membrane and expressed in roots, facilitates the transport of lead, Zn, and Cu. Additionally, *OsHMA2* and *OsHMA5*, located at the plasma membrane, play a crucial role in translocating heavy metals from roots to shoots [157].

In monocotyledonous crops, only a few Cu transporters have been identified and characterized, such as seven *COPT* proteins and *OsYSL16*-Cu-nicotiamine transporter [158] in rice and Cu transporters viz. *ZmCOPT1, ZmCOPT2*, & *ZmCOPT3* in maize [159]. To date, only one study has been published, where researchers reported the identification of a copper transporter in wheat [160]. This study unveiled a novel golgi-localized copper transporter gene family, *TaCT1*, in common wheat. The researchers isolated three *TaCT1* homoeologous genes and assigned them to group 5 chromosomes. Each *TaCT1* gene (*TaCT1-5 A*, *TaCT1-5B*, and *TaCT1-5D*) features 12 transmembrane domains, categorizing it as part of the major facilitator superfamily (MFS) [160]. This identification positions *TaCT1* as a distinct member of the MFS family, different from the two known types of copper transporter genes [160].

Genes/TFs involved in the uptake and translocation of different minerals are promising targets to improve nutrient remobilization in the wheat grains, which can be helpful to mitigate Zn, Fe, Se and Cu deficiencies that afflict many regions of the developing world [17, 161].

### Mechanisms of mineral accumulations in grains

Accumulating metals like Zn, Fe, Se and Cu is a complex physiological trait governed by the cumulative expression of gene for uptake, transport, distribution and sequestration in different plant parts. Metals are deposited within the grain by means of HMAs, and CDFs, often referred to as MTPs and NRAMPS [162]. Zn concentration varies between organs, tissues and intracellular compartments within a plant system. This divergence arises due to the differential expression of metal transporter proteins (e.g., *MTPs*, *ZIPs*, *VITs* etc.) and intracellular binding sites in a particular organ [161]. Seed has tissues like embryo, endosperm and aleurone, which are covered by maternal tissues (seed coat). A single vascular trace connects the developing seed to the maternal plant [11]. The embryo and endosperm are not symplastically connected to the vascular bundle that ends at the seed coat. Unloading of nutrients comes from roots system through the phloem to seeds, the all areas of the plants are covered by a continuous network of water-conducting channels made up of the xylem, vessels, and tracheids of the roots, stems, and leaves. Water and soluble mineral nutrients are transported throughout the plant by this system from the roots. Plants possess suberized cells, such as cork, in their outermost tissues. These cells play a vital role in regulating temperature and water loss, as well as serving as a protective barrier from environmental infections and aggressors. The nutrients delivered by phloem allocated in surrounding maternal tissue are usually effluxes into apoplastic space that bifurcates the maternal and filial tissues. Remobilization of minerals from the leaves to the grain occurs at the time of uptake of these micronutrients from the root to the shoot. However, micronutrients are mobilised by xylem and phloem for their transport to grain as well as other vegetative parts of the plant [163, 164].

Micronutrients are deposited in two important locations within the seed: ferritin for Fe and the vacuole for both Zn and Fe. Ferritin is a widely distributed protein that acts as a storage site for Fe. In case of limited supply, Fe is released from ferritin [164]. Therefore, ferritin plays a crucial role in maintaining Fe homeostasis in plants, including cereals like wheat. The presence of free Fe (if not stored in ferritin) can lead to the formation of free radicals, which can cause damage to the plant. It has been observed that only about 5% of the total Fe is stored in the form of ferritin, while the remaining 95% is stored in vacuoles. The primary function of ferritin and aleurone in seeds is not storage but rather protection of the plant from oxidative stress [165, 166]. On the other hand, phytic acid functions as an antinutritive agent by inhibiting the absorption of essential minerals, including Fe and Zn [167]. Studies in rice have shown that overexpression of ferritin cDNA derived from soybean specifically in the endosperm can significantly increase Fe content in the grains, sometimes up to two to three times [167, 168]. However, attempts to achieve a similar increase in Fe content in leaves through excessive transport from the roots have not been successful, as the leaves appeared to be depleted of Fe due to its transport to the seeds [169]. In wheat, extensive research has been conducted on genes related to ferritin, vacuole transporter-like proteins (*VTLs*), and vacuole transporters (*VITs*). Among the ferritin genes, *TaFer1* (homoeologous group 5) and *TaFer2* (homoeologous group 4) have been identified as differentially regulated and expressed. The TaFer1 and TaFer2 genes are responsible for encoding two distinct isoforms of ferritin, which are likely to have diverse functionalities and exhibit heteropolymer structures in cereals. It is feasible to implement iron biofortification in wheat grains. The intragenic overexpression of the *TaFer1-A* gene in the endosperm leads to a significant increase in the iron concentration inside the grain, ranging from 50 to 85% [170]. Wheat also possesses VIT genes, including *TaVIT1*, *TaVIT2*, and *TaVIT3* (homoeologous groups 5, 2, 7), as well as VTL genes, such as *TaVTL1*, *TaVTL2*, *TaVTL4*, and *TaVTL5* (homoeologous groups 2, 4, 6) [171, 172]. According to Kenzhebayeva et al. [112], the *VIT2* is involved in the intracellular transport and storage of iron. Similar to *Arabidopsis*, it has been reported that vacuolar transporters encoded by these genes play a crucial role in storing approximately 95% of Fe in vacuoles [173]. Furthermore, the expression levels of all VIT genes were found to be particularly high in the aleurone layer and lower in the endosperm, exhibiting a strong correlation with Fe abundance in the aleurone [171]. Specifically, *TaVIT2* showed higher expression levels compared to *TaVIT1*. Transgenic studies using *TaVIT2* with an endosperm-specific strong promoter namely *GLUID-1*, resulted in a twofold increase in Fe content without a concurrent increase in phytate, which can negatively affect bioavailability of Fe. When *TaVIT2* was combined with the *GLU-1D-1* promoter, which is derived from the endosperm excluding the aleurone, transgenic lines exhibited a more than twofold increase in Fe content in white flour [171].

The aforementioned information regarding VITs and VTLs highlights the potential benefits of enhancing vacuolar storage Kenzhebayeva et al. [112] rather than ferritin storage in cereal grains. Unlike ferritin genes, which are not expressed in the endosperm, vacuolar storage is the typical mode of storage in cereal grains [112]. This is particularly important because the aleurone layer is often lost or discarded during the milling process of wheat grains, leading to a reduction in the nutritional quality of the end product. Therefore, it is evident that *VIT* and *VTL* genes play a significantly more important role than ferritin genes in increasing the storage capacity of wheat grains [17]. Another relevant gene in wheat for biofortification is the major grain protein gene (*Gpc1*), which also influences the content of Zn and Fe in the grain. This gene, derived from tetraploid wheat, is mapped on chromosome 6B and encodes a NAC transcription factor known as *NAM-B1*. Due to its substantial influence on grain protein and mineral content, as well as its pleiotropic effects on senescence rate and grain size, the *NAM-B1* gene in wheat has been the subject of intense research and application in breeding programmes for almost three decades [174]. *NAM-B1* promotes senescence and is responsible for nutrient remobilization from leaves to grains [175, 176]. Numerous studies in wheat have demonstrated that introgression of *Gpc-B1* leads to improvements in Zn and Fe content in the grain as well as an enhancement in GPC [177]. During the early stage of senescence, the *Gpc-B1* gene regulates the expression of various genes, including transporters of the ZIP and YSL families These gene families facilitate the movement of Zn and Fe from the cytoplasm into the phloem and genes involved in the biosynthesis of chelators, which aid in the phloem-based transport of Zn and Fe to the grains [178, 179]. Therefore, the *Gpc-B1* gene exerts a significant impact on Zn and Fe content and can be exploited for biofortification of Zn and Fe in wheat grains. The correlation analysis indicated that GCuC correlated positively with GZnC. As a result, the accumulation of Cu and Zn in grains could not be independent of one another. Thus it is hypothesised that zinc and copper in wheat grains may be synergistic. The genotypic variations in the accumulation of zinc and copper in wheat grain are poorly understood [180].

According to Wang et al. [143], the presence of two selenium species, namely selenomethionine and selenocysteine, has been detected in wheat grains. Typically, plants can accumulate selenium species within several subcellular compartments. Ding et al. [181] found that selenium (Se) exhibited a predominant concentration inside the cell wall and cytosol fractions of paddy rice. Sabbioni et al. [182] demonstrated the presence of selenium (Se) in various subcellular fractions, with a particular emphasis on the cytoplasm of microalgae, as well as the membranes and organelles of spring tea leaves [183]. The precise subcellular distribution of selenium (Se) in wheat remains unclear [137].

### Different approaches for wheat biofortification

**Fig. 2:**
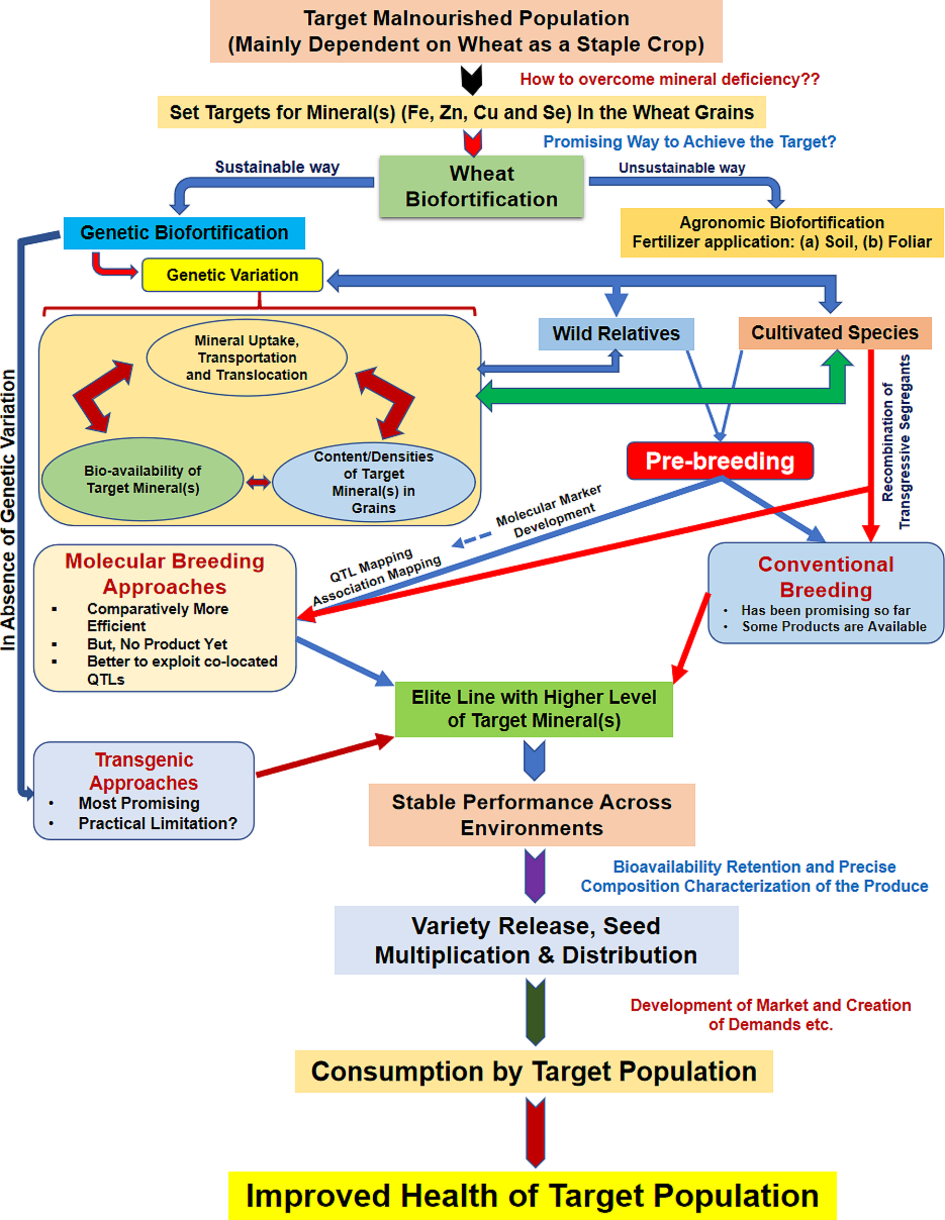
Different approaches for wheat biofortification

Biofortification strives to augment the accessibility of vital elements in the edible components of crops. When it comes to wheat biofortification, four primary approaches are employed (Fig. 2). Moreover, the integration of metabolic engineering and CRISPR technology presents further opportunities. Metabolic engineering involves the modification of metabolic pathways to amplify the synthesis, accumulation, and transport of nutrients. These methods not only complement conventional biofortification techniques but also open up novel pathways to elevate the nutritional profile of wheat.

### Conventional breeding for wheat biofortification

This is the widely accepted, sustainable, cost-effective, and non-transgenic approach for biofortification. It involves crossing parents with high nutrient levels with a recipient parent deficient in nutrients but having desirable agronomic traits and selecting plants with both improved micronutrients and desired agronomic traits [19, 184]. Breeding strategies primarily focus on transferring genes for Fe and Zn homeostasis from wild relatives, such as *Triticum spelta-*derived synthetics and *Triticum dicoccum* landraces, into elite wheat genotypes [18]. International organizations, like the HarvestPlus program, are actively involved in increasing nutrient content in crops, including Fe, Zn, and pro-vitamin A (beta-carotenoid), in various crops such as rice, wheat, maize, beans, and sweet potato [1]. Several biofortified wheat varieties, including MACS 4058, HD 3298, HI 1633, DBW 303, PBW752, PBW757 [additional file 3 (Table S3)], have been released in India [1]. Pakistani wheat breeders have also developed a Zn- and Fe-fortified wheat variety named ‘Akbar-2019’ through conventional breeding in collaboration with CIMMYT under the HarvestPlus program [185https://www.ilri.org/news/cgiar-research-program-agriculture-nutrition-and-health-publishes-2020-annual-report accessed on 16 October 2022; https://a4nh.cgiar.org/annual-report-2020/ accessed on 16 October 2022]. Numerous research papers have been published on increasing Fe and Zn content in wheat using the breeding approach [30]. However, this approach has limitations, including limited genetic variation for micronutrients in the plant gene pool and the extensive time and effort required for multiple breeding cycles [17].

### Agronomic approaches for wheat biofortification

In this approach, the nutritional quality of food crops is enhanced through the physical application of nutrients, which involves the process of solubilization and mobilization of micronutrients from the soil to the edible parts of cereals or application directly to the leaves of the crop (foliar application) [184, 186]. Fertilizers can be applied either to the soil or directly to the foliage of crops to increase the availability of micronutrients in the grains. This method is considered convenient, cost-effective, and user-friendly compared to other approaches. However, it is important to note that this approach can have negative environmental impacts and requires careful attention. Di et al. [187] reported the biofortification of wheat with Se through foliar spray application (15 g/ha) of Se. More recently, Jalal et al. [188] demonstrated Zn biofortification in wheat by employing the inoculation of *P. fluorescens* and *B. subtilis* along with foliar nano-Zn application as a sustainable method to enhance the nutritional value of wheat. Notably, this approach has shown promising results in improving the grain quality of wheat (Additional file 2; Table S2).

### Transgenesis for wheat biofortification

This approach is currently underutilized, despite extensive research being conducted using this method [189]. In genetic engineering, desirable genes can be transferred between plants regardless of their evolutionary or taxonomic relationship, allowing for the production of transgenic crops (Additional file 3; Table S3). This approach was first employed in wheat to increase grain Fe content by ectopically overexpressing the wheat *ferritin* gene [170]. However, this gene was found to be unstable and not transmitted to the next generation (Borg, Personal communication). In wheat, bacterial genes such as Phytoene synthase (PSY) and carotene desaturase have been utilized to enhance the content of beta-carotene (pro-vitamin A) [190]. Studies have reported the overexpression of rice gene *OsNAS2* in wheat, resulting in 40–100% grain Fe levels and 60–250% gain in Zn level [23, 191]. However, this approach faces limitations such as the time, cost, and effort required for research, low acceptance by farmers, and the regulatory processes that impose significant expenses and time constraints [23].

### Molecular breeding approaches for wheat biofortification

This approach addresses the limitations of conventional breeding, particularly when dealing with polygenic variations of micronutrients, which can be challenging to improve through traditional breeding methods [36]. In recent years, significant advancements have been made in MAS and the identification of genomic regions associated with micronutrient contents in wheat. Previous studies have utilized QTL mapping and GWAS to identify these regions, as discussed in earlier sections.

### Mutagenesis for creating novel genetic variation

Mutagenesis stands as a potent tool, capable of inducing genetic variation in germplasm lacking inherent genetic diversity for the trait in question [17]. Induced mutagenesis, a robust technique within plant breeding, contributes to the creation of ‘smart’ crop varieties, thus bolstering nutritional and global food security agendas [192]. The mutagenesis has led to the development of over 3,320 varieties belonging to around 214 plant species, as documented within the IAEA/FAO database (https://mvd.iaea.org/ accessed on 15 November 2022). Out of 3320 varieties 276 are wheat varieties. However, scant reports exist concerning mutants that exhibit enhanced Fe, Zn, Cu, and Se contents in grains of wheat and other cereals. So far, only three studies have been reported [56, 193], generating stable mutants with improved grain parameters such as length, width, quality, and area, underscoring the intricate interplay between genetic variation of micronutrients and grain traits, a pivotal insight [56, 193]. In another study, M5 mutant lines of spring wheat were developed through gamma-irradiation that had lower phytic acid content, enhanced Fe bioavailability, and higher grain Fe and Zn content as compared to the parent variety [112]

### Metabolic engineering for wheat biofortification

Metabolic engineering has emerged as a sustainable, effective, and cost-efficient method in crop biofortification [194]. This approach involves the genetic modification of metabolic pathways to enhance the nutritional value of crops. By employing push and block strategies, such as the overexpression of *CrtB* and the silencing of *TaHyd*, a significant accumulation of β-carotene was achieved in wheat, with levels reaching up to 5.06 µg/g, representing a 31-fold increase [195]. Moreover, by co-expressing the soybean *Gm8gGCHI* and tomato *LeADCS* genes under the regulation of the wheat endosperm-specific promoter 1Dx5, transgenic wheat grains demonstrated a 5.6-fold increase in folate content [196]. These examples highlight the potential of metabolic engineering for enhancing crop nutritional composition.

### Genome editing for wheat biofortification

The CRISPR/Cas system was introduced in rice and wheat in 2013, revolutionizing genetic modification [197]. This technology offers a promising avenue for directly modifying biofortification-related genes in elite wheat cultivars, bypassing the time-consuming process of conventional breeding [198]. To effectively utilize CRISPR/Cas technology for biofortification in wheat, a comprehensive understanding of the genes that regulate Fe, Zn, Cu and Se content is crucial. Wang et al. [199] conducted multiplexed genome editing in hexaploid wheat, revealing the frequency of mutations and their heritability [113]. Furthermore, Ibrahim et al. [200] achieved a significant breakthrough by disrupting the *TaIPK1* gene using CRISPR/Cas, resulting in reduced phytic acid levels and enhanced Fe and Zn contents in wheat grains [201]. These advancements underscore the potential of CRISPR/Cas technology for precise and targeted modifications to improve wheat biofortification.

### Prospects of accelerating wheat biofortification through speed breeding

Traditional crop breeding methods are time-consuming, often taking several years before a new variety is released. To overcome this challenge and expedite the breeding process, speed breeding has emerged as a game changer [201]. Speed breeding involves precise control of temperature, light intensity, and daytime length to enhance photosynthesis rates, leading to early flowering and annual seed harvesting, thereby reducing generation time and enabling rapid phenotyping of multiple traits [202]. This innovative approach has been successfully implemented in various crop species, including wheat, using artificial lighting within controlled environment growth chambers [89, 203]. By combining speed breeding with CRISPR/Cas technology, the biofortification of wheat and other crops can be further advanced, offering promising prospects for enhancing nutritional quality.

### Challenges, limitations, and opportunities in wheat biofortification

Despite improved resources, wheat biofortification encounters several challenges [7] including the need for consensus in genetics and genomics research through a shared reference genome sequence and standardized nomenclature. This harmonization would facilitate the optimal utilization of existing research findings. Incorporating reference sequences for diverse wheat genotypes adds complexity, but leveraging multiple reference sequences, or pangenomes, offers significant advantages. Further, developing a comprehensive super-pangenome for the entire *Triticum* genus may hold potential to revolutionize wheat biofortification.

Moreover, many programs enhancing wheat nutrient content face challenges due to expensive, labour-intensive phenotyping methods [7]. Furthermore, the nutrient bioavailability stands as a pivotal determinant of grain quality [17]. The collaboration of wheat scientists and nutritionists becomes critical in determining the nutrient bioavailability of biofortified crops through comprehensive in vitro investigations and human intervention experiments. In addition, genetic transformation for biofortification brings challenges, especially in navigating regulatory landscapes for genetically modified organisms. Addressing these challenges may be vital for successful wheat biofortification.

Further, the success of biofortified wheat hinges upon the acceptance of consumers and the demand within the market. Therefore, it is imperative to raise awareness about the multitude of benefits that biofortified crops offer and effectively address any misconceptions or concerns that may arise. An essential aspect to consider is the alignment of biofortified wheat varieties with consumer preferences in terms of taste, appearance, cooking qualities, and sensory attributes. This requires a thorough understanding of consumer needs and desires, which can be attained through targeted breeding efforts and comprehensive sensory evaluations. However, the task of achieving widespread adoption and availability of biofortified wheat among the intended populations presents distinct challenges. This entails establishing robust systems for seed multiplication and distribution, providing comprehensive training and capacity-building for farmers, and fostering collaboration with stakeholders along the agricultural value chain. Currently, there is a lack of incentives and motivation for farmers to engage in cultivating improved crops, which impedes progress in biofortification efforts. Moreover, consumers often lack awareness about identifying and accessing high-quality products from biofortified crops, further hindering their acceptance. Overcoming these hurdles requires a focused, coordinated effort, including policy implementation incentivizing biofortified wheat adoption by farmers, enhancing market access and infrastructure, and conducting extensive consumer education and awareness initiatives.

Moreover, substantial investments in biofortification programs, reinforced collaborations, and active promotion of biofortified crops stand as imperative actions to tackle global nutritional challenges and ensure their extensive adoption and impact. Opportunities in biofortification include speed breeding techniques for rapid development of nutrient-rich wheat varieties, multi-biofortification for comprehensive nutrient enhancement, nutrigenetics to understand genetic influences on nutritional composition, and nutrigenomics for personalized nutrition and tailored dietary interventions. Further, tissue culture-based methods might provide powerful tools for wheat biofortification, enabling the selection, manipulation, and combination of desirable traits to develop improved wheat varieties with enhanced nutritional value. Additionally, by placing emphasis on investment, collaboration, awareness, and innovative methods such as speed breeding, multi-biofortification, nutrigenetics, and nutrigenomics, we can elevate nutritional security and enhance global health. These strategies may effectively address the intricate dilemmas of malnutrition and advance well-being on a global scale.

### Concluding remarks and future perspectives

Micronutrient malnutrition poses a significant global challenge, with wheat being a primary staple crop second only to maize and rice. Given the heavy reliance on wheat as a major energy source, the low levels of micronutrients in most wheat varieties worldwide necessitate the implementation of wheat biofortification strategies for enhancing micronutrient concentrations. However, several challenges hinder the progress of wheat biofortification, including fragmented research efforts conducted independently across the globe. To overcome these challenges, researchers must establish collaborative consortiums with shared goals and objectives. The integration of omics approaches such as genomics, metabolomics, phenomics, and transcriptomics can provide comprehensive insights and address critical barriers to improving the nutritional quality of wheat.

### Electronic supplementary material

Below is the link to the electronic supplementary material.

Supplementary Material 1

Supplementary Material 2

Supplementary Material 3

## Data Availability

Genetic variation in wheat for iron, Zinc, copper and selenium in mg/kg (Additional file 1: Table S1). List of QTLs reported for micronutrients (Zn, Fe and Se) in wheat (Additional file 2; Table S2). The Different approaches used for the biofortification of micronutrients in wheat are accessible in the supplemental table (Additional file 3; Table S3).
