# Supplementary material for: Biofortification of Triticum species: a stepping stone to combat malnutrition
Source: BMC Plant Biol. 2024 Jul 15;24:668. doi: 10.1186/s12870-024-05161-x (PMC11247745; doi:10.1186/s12870-024-05161-x)
Supplement: Supplementary file 3 — Supplementary Material 3 [file 12870_2024_5161_MOESM3_ESM.docx]

| Table S3: Different approaches used for biofortification of micronutrients in wheat | | | |
| --- | --- | --- | --- |
| **Approach** | **Research/variety released** | **Biofortification type** | **Reference/Source** |
| Conventional breeding approach | India: BHU 1, BHU 3, BHU 5, BHU 6, BHU 17, BHU 18 Pakistan: NR 419, 42, 421, Zincol | Zinc ,Iron | CIAT, CIMMYT, HarvestPlus (https://www.harvestplus.org/wp-content/uploads/2014/09/Biofortification_Progress_Briefs_August2014_WEB_2_0.pdf) |
|  | WB2(India) | Zinc and iron | Indian Institute of Wheat and Barley Research, India |
|  | HI 8759(India) | Zinc , iron, protein | ICAR-Indian Agricultural Research Institute IARI,India |
|  | HPBW 01(India) | Zinc and iron | Punjab Agricultural University, India |
|  | HI 1605(India) | Zinc and iron | ICAR-IARI, Regional Station, Indore, Madhya Pradesh. |
|  | MACS 4028(India) | Zinc, iron and protein | Agharkar Research Institute, Pune, Maharashtra, |
|  | HI 8627(India) | Carotene | Indian agricultural research institute,India |
|  | HD 3171(India) | Zinc | Indian agricultural research institute,Delhi,India |
|  | HI 8777 | Zinc and iron | ICAR-Indian Agricultural Research Institute, Regional Station, Indore |
|  | PBW 752 | Protein | Punjab Agricultural University, Ludhiana under ICAR-All India Coordinated Research Project on Wheat & Barley |
|  | PBW 757 | Zinc | Punjab Agricultural University, Ludhiana under ICAR-All India Coordinated Research Project on Wheat & Barley |
|  | UAS 375+D14:D26 | Iron | ICAR-Indian Institute of Wheat & Barley Research, Karnal |
|  | DBW 173 | Protein and iron | ICAR-Indian Institute of Wheat & Barley Research, Karnal |
|  | UAS 375 | Protein | University of Agricultural Sciences, Dharwad under ICAR-All India Coordinated Research Project on Wheat & Barley |
|  | DDW 47 | Protein and iron | ICAR-Indian Institute of Wheat & Barley Research, Karnal |
|  | PBW 771 | Zinc | Punjab Agricultural University, Ludhiana under ICAR-All Indian Coordinated Research Project on Wheat & Barley |
|  | HI 8802 (durum) | Protein | ICAR-Indian Agricultural Research Institute, Regional Station, Indore |
|  | HI 8805 (durum) | Protein and iron | ICAR-Indian Agricultural Research Institute, Regional Station, Indore |
|  | HD 3249 | Iron | ICAR-Indian Agricultural Research Institute, New Delhi |
|  | MACS 4058 (durum) | Zinc, iron and protein | Agharkar Research Institute, Pune under ICAR-All India Coordinated Research Project on Wheat & Barley Year |
|  | HD 3298 | Protein and iron | ICAR-Indian Agricultural Research Institute, New Delhi |
|  | HI 1633 | Zinc, iron and protein | ICAR-Indian Agricultural Research Institute, Regional Station, Indore |
|  | DBW 303 | Protein | ICAR-Indian Institute of Wheat & Barley Research, Karnal |
|  | DDW 48 (durum) | Protein | ICAR-Indian Institute of Wheat & Barley Research, Karnal |
|  |  |  |  |
|  | Research | Zinc and iron | Cakmak et al. 1999; Monasterio and Graham 2000; Welch et al. 2005; Cakmak et al. 2004 |
|  | Research | Lutein | Digesu et al. 2009; Ficco et al. 2014 |
|  | China: Black-grained wheat | Anthocyanins (colored wheat) | Havrlentova et al. 2014 |
|  | Austria: Indigo |  | Havrlentova et al. 2014 |
|  | Registered/Research:NABIMG-9, NABIMG-10, NABIMG-11 (India) | Anthocyanin (colored wheat) | Garg et al. 2016 |
| Agronomic approach | Research | Iron | Aciksoz et al.2011 |
|  | Research | Zinc | Cakmak et al. 2010; Yang et al. 2011 |
|  | Research | Zinc | Zhang et al. 2012 |
|  | Research | Se | Aro et al. 207 |
|  | Research | P fertilizer + mycorrhiza | Noori et al.2014 |
|  | Research | Organic + chemical fertilizers (iron) | Ramzani et al. 2016 |
|  | Research | Bacillus aryabhattai (zinc) | Ramesh et al. 2014 |
|  | Research | Iron and Zinc | Ramzan et al. 2020 |
|  | Research | Zn-CNP nanocarrier | Dapkekar et al. 2018 |
|  | Research | Se | Di et al. 2023 |
|  | Research | Zn | Jalal et al. 2023 |
| Transgenic approach | Research | Provitamin A Carotenoids | Wang et al. 2014; Cong et al. 2009 |
|  | Research | Iron | Sui et al. 2012; Borg et al. 2012 |
|  | Research | Iron | Beasley et al., 2019 |
|  | Research | Iron | Connorton et al. 2017 |
|  | Research | Iron | Singh et al. 2017 |
|  | Research | Phytase or phytic acid | Brinch-Pedersen et al. 2000; Bhati et al. 2016 |
|  | Research | Amino acid composition | Tamás et al. 2009 |
|  | Research | Anthocyanin | Doshi et al. 2006 |
|  | Research | Amylose content | Sestili et al. 2010 |
